# Factors Associated With Self‐Efficacy in Medical Staff: A Cross‐Sectional Study

**DOI:** 10.1002/hsr2.71124

**Published:** 2025-07-30

**Authors:** Dara Rasoal, Zahra Karimian, Zahra Javanbakht, Kamran Vafaee, Mina Abbasi, Arezoo Haseli

**Affiliations:** ^1^ School of Health and Welfare Dalarna University Falun Sweden; ^2^ Department of Midwifery Kashan University of Medical Sciences Kashan Iran; ^3^ Clinical Research Development Center, Motazedi Hospital Kermanshah University of Medical Sciences Kermanshah Iran; ^4^ Clinical Research Development Center, Imam Reza Hospital Kermanshah University of Medical Sciences Kermanshah Iran; ^5^ Nursing Department, Nursing and Midwifery School Kermanshah University of Medical Sciences Kermanshah Iran; ^6^ School of Nursing and Midwifery Zanjan University of Medical Sciences Zanjan Iran; ^7^ Family Health and Population Growth Research Center, Health Policy and Promotion Research Institute Kermanshah University of Medical Sciences Kermanshah Iran

**Keywords:** anxiety, health personnel, self‐efficacy, sleep quality, social support

## Abstract

**Background and Aims:**

Self‐efficacy is essential for medical staff to manage challenges and maintain professional commitment. In Iran, high workloads, staff shortages, and limited mental health support may negatively affect self‐efficacy and well‐being. This study examines key factors influencing self‐efficacy among Iranian medical staff.

**Methods:**

The survey included 411 Iranian medical staff in July 2024. The sample size was determined based on power analysis to ensure sufficient statistical power for detecting significant associations between self‐efficacy and its related factors. Participants completed Pittsburgh Sleep Quality Index (PSQI), General Health Questionnaire (GHQ), General Self‐Efficacy Scale (GSE), and Social Support Indexes (SSI). Data were analyzed by descriptive statistics and analytical tests (*χ* ^2^, Pearson, correlation coefficient, and logistic regression) at a significance level less than 0.05.

**Results:**

The mean (SD) of general self‐efficacy of staff was at a moderate level (22.55 ± 5.40). The mean (SD) of PSQI and SSI was 7.67 ± 4.46 and 29.80 ± 13.94, respectively, which indicates poor sleep quality and moderate social support. The mean (SD) of the general health of staff was 51.48 ± 03, which indicates that the general health of workers is at risk. Self‐efficacy score was directly related to higher sleep quality (OR: 4.362, CI: 1.481–12.853, *p* = 0.008), general health quality physically (OR: 2.103, CI: 1.041–4.248, *p* = 0.038), less anxiety of general health quality (OR: 2.175, CI: 2.203–3.934, *p* = 0.010), and general social support (OR: 7.099, CI: 3.121–16.149, *p* < 0.001).

**Conclusion:**

This study identified poor sleep quality, moderate self‐efficacy, low social support, and general health risks among Iranian medical staff. Given the positive association between self‐efficacy and various well‐being indicators, targeted interventions—such as mindfulness training, sleep hygiene programs, and social support initiatives—are recommended. Strengthening self‐efficacy through institutional support and stress management strategies should be integrated into healthcare policies to promote a more resilient and effective workforce.

## Summary

- What's known
  ◦ Multiple factors can influence self‐efficacy in medical staff.
- What's new
  ◦ Self‐efficacy increases with high sleep quality, general physical health, low anxiety, and high social support.
  ◦ General self‐efficacy is a result of physical and mental health and social support.
  ◦ The general self‐efficacy of Iranian medical staff was at a moderate level.
- What are the clinical implications
  ◦ Increasing the self‐efficacy of employees should be taken into consideration in patient care improvement programs.

## 1 Introduction

Healthy medical staff with high levels of self‐efficacy can provide optimal health care to patients [1, 2]. General self‐efficacy (GSE) is the belief in one's ability to accomplish difficult or new tasks and cope with adversity arising from particular difficult situations [3]. It's generally defined as a judgment of “how well one can perform actions necessary to cope with future situations” [4] and helps the medical staff to adapt to high‐risk and stressful work, and helps them maintain a stable mental state. The relationship between general health and sleep quality with self‐efficacy has been shown by previous studies [5, 6, 7]. People with high levels of self‐efficacy can more effectively reduce potential health problems and achieve more meaningful outcomes. Job stress can lead to decreased self‐efficacy, which can lead to decreased work quality [8].

Various factors are associated with general self‐efficacy, including sleep quality [9], psychological issues, and social support [9]. Self‐efficacy plays a crucial role in an individual's ability to overcome challenges and is closely linked to sleep. Sleep deprivation among employees has several negative consequences, such as reduced work performance, strained workplace relationships, decreased organizational citizenship behavior, and an increase in unethical workplace conduct [5, 6]. Social support is another key factor influencing self‐efficacy. Studies indicate a positive correlation between self‐efficacy and the level of social support an individual receives. As an essential external resource, social support has gained increasing attention from researchers in various fields, both domestically and internationally [10].

According to Bandura et al., self‐efficacy plays a pivotal role in the self‐regulation of affective states [11]. Research indicates that job stress negatively impacts health, reduces overall well‐being, and lowers efficiency [12]. Additionally, social support has been consistently recognized as a moderating factor in maintaining general health [13]. Examining the self‐efficacy of medical staff and its related factors is essential for promoting their well‐being and ensuring optimal patient care. This study employs a multivariate analysis to assess the effects of social support, sleep quality, and general health on the self‐efficacy of Iranian medical staff.

## 2 Materials and Methods

### 2.1 Participants and Procedure

The study is part of a broader research project named 'Health Status in Medical Staff'. The survey was done in July 2024. The study group consisted of 411 staff from all government hospitals in Ilam, Iran (four hospitals with more than 5000 medical staff without physicians). Healthcare workers included nurses, midwives, anesthetists (nurses), surgical technologists, nursing aids, and paramedics. Descriptive and working characteristics of the study participants are described in Table 1.

**Table 1:** Demographic and working data of the medical staff.

Categorized variable	Sample size (*N *= 411)	Number	%
Gender			
Males	411	87	21.17
Females		324	78.83
Education			
Master's degree or above	411	84	20.44
Bachelor's degree		256	62.29
Associate degree		58	14.11
Diploma		13	3.16
Marital status			
Single	406	143	35.22
Married		262	64.78
Profession			
Nurse	411	296	72.02
Midwife		44	10.71
Anesthetist (nurse)		25	6.08
Surgical technologist		28	6.81
Nursing aids		13	3.16
Paramedic		5	1.22
Continuous variables	Sample size (*N* = 411)	Range	Mean ± SD
Staff's age (year)	408	23–54	33.31 ± 4.81
Working experience (years)	411	1–27	11.08 ± 5.66

Since most healthcare staff with fixed morning shifts hold managerial roles or do not provide direct nursing care (e.g., infection control nurses, complaints nurses), and do not stay overnight in the hospital, we included only those with rotating shifts in the hospital's clinical wards in this study. Other inclusion criteria included healthcare workers with any amount of work experience, and any job role, with no medical history or underlying chronic mental illness, and no serious mental trauma in the past 2 months.

To respect participants' autonomy, responding to the questions was voluntary. However, questionnaires with more than 20% of items left unanswered were excluded from the study.

Based on the means and standard deviations of variables from previous studies [14, 15, 16, 17] and then calculating the sample size separately, the maximum sample size using the following formula was estimated to be 386 people.

$$n=Z^{2}\frac{{\left(\mathrm{SD}\right)}^{2}}{d^{2}}$$

Convenience sampling was used for this study. As each hospital served specific patient groups (e.g., women, internal medicine, general medicine), sampling was conducted across all hospitals. Given the research population (over 5000 individuals) and the required sample size (386), and accounting for the potential exclusion of up to 10% due to incomplete responses, the research team decided to randomly select 10% of personnel from each department using a simple random sampling method. Those who met the inclusion criteria were invited to participate.

First, in coordination and collaboration with hospital nursing department managers, we received a list of medical staff and phone numbers of them. Participants were contacted and informed about the study's objectives. Inclusion criteria were assessed, and those willing to participate received a link to the online questionnaire. The data collection process included the following steps: uploading consent forms and the questionnaire to the online platform; receiving the questionnaire link from the platform; sending the link to eligible participants; participants completing the questionnaire online; automatic transfer of responses to an Excel file; exporting the data to SPSS software; removing incomplete responses; filtering the data; and finally, analyzing the results.

Participants who did not submit their responses within 24 h received up to two reminder messages, sent at 24 and 48‐h intervals after the initial invitation. In total, the questionnaire was distributed to 517 medical staff. Of these, 419 completed it (response rate = 81%). Eight questionnaires with more than 20% of unanswered items were excluded, resulting in a final sample of 411 participants.

## 3 Assessment Tools

### 3.1 General Self‐Efficacy Scale

The General Self‐Efficacy Scale (GSE), developed by German psychologist Schwarzer [18], includes 10 items. Its scoring is based on a four‐point Likert scoring. In this order, the range of points will be between 10 and 40. Scores between 10 and 20 are considered low self‐efficacy, scores between 21 and 30 as medium self‐efficacy, and scores above 31 as high self‐efficacy [19]. The reliability of the Persian version of this scale was previously established by Carpenter and Andrykowski [20], who reported a Cronbach's alpha of 0.83. The questionnaire's reliability and validity had already been confirmed. In the present study, internal consistency was also assessed using Cronbach's alpha, yielding a value of 0.81.

### 3.2 Pittsburgh Sleep Quality Index

PSQI is a self‐report questionnaire that assesses sleep quality and disturbances in the last month. Each dimension is rated in the range 0–3, with an overall score in the range 0–21, with higher values indicating poorer sleep quality [20]. We considered a PSQI global score > 5 as poor sleep quality and less than 5 as good sleep quality

We considered a PSQI global score > 5 as poor sleep quality and less than or equal to 5 as good sleep quality [21]. The Cronbach's *α* value was 0.83 for the original PSQI equipment, and 0.79 for the Iranian version of PSQI [22].

### 3.3 Social Support Indexes

We used the Social Support Index (SSI) to measure the type and level of social support. The index consists of 12 items in three categories: family, friends, and others, with a total score ranging from 7 to 56. Higher scores indicate higher levels of social support [23]. The internal consistency of Cronbach's alpha using the SSI was 0.798.

### 3.4 General Health Questionnaire

To examine psychological well‐being, we used the General Health Questionnaire‐28 (GHQ‐28). The GHQ‐28 has four subscales for somatic symptoms, anxiety/insomnia, social dysfunction, and severe depression, each containing seven items.

The scoring method in this questionnaire is from 1 to 4 for options A to D, respectively. The subject's scores in each subscale can be a minimum of 4 and a maximum of 28. A score higher than 14 shows the probability of a problem is higher. The cut‐off score for the total score is 23. Therefore, a total test score higher than 23, the subject's general health is more dangerous. The validity and reliability of this scale has been proven in studies [24].

### 3.5 Statistical Analysis

We evaluated frequencies, percentages, means, standard deviations, and correlations between variables.

Multiple logistic regression analyses were performed separately to evaluate factors associated with self‐efficacy in medical staff. In regression analysis, we classified each variable into two groups of zero or one. Thus, poor sleep quality, poor/moderate social support, very low/low general health, and low self‐efficacy were classified as zero, and good sleep quality, high social support, moderate/high general health, and high self‐efficacy were classified as one. We used SPSS 18 [25] for all calculations at a significance level of less than 0.05.

## 4 Ethical Considerations

This study was a research project approved by the Ethics Committee of the Deputy for Research at Kermanshah University of Medical Sciences, Kermanshah, Iran (Approval Code: IR.KUMS.REC.1403.183). All participants were fully informed about the purpose and procedures of the study and provided both oral and written informed consent before participation.

## 5 Results

The aim of this study was to identify factors associated with self‐efficacy among Iranian medical staff. This section presents the findings on general self‐efficacy, general health, sleep quality, and social support among medical professionals. Descriptive statistics, including mean scores and standard deviations for each variable, provide an overview of the participants' well‐being and self‐efficacy levels. Additionally, the distribution of sleep quality across different medical professions is analyzed to highlight potential concerns regarding workplace health. These findings offer insights into the relationships between these factors, which may have implications for staff performance and the quality of patient care.

The mean score of the General Health Questionnaire suggests that the general health of the staff is at risk. Since a value above 14 for each subscale indicates potential health concerns, it can be inferred that employees may experience issues, particularly in terms of physical health and anxiety levels. Findings also indicate that poor sleep quality (PSQI > 5) was prevalent among different professional groups, affecting 83.8% of nurses, 32.4% of midwives, 40% of anesthetists, 46.43% of surgical technologists, 69.23% of nursing aides, and 60% of paramedics.

The mean (SD) scores for General Self‐Efficacy (GSE), General Health Questionnaire (GHQ), Pittsburgh Sleep Quality Index (PSQI), and Social Support Index (SSI) were 22.546 (5.403), 51.487 (8.034), 7.67 (4.98), and 29.8 (13.94), respectively. The general self‐efficacy of the staff was found to be at a moderate level. The subscales of the questionnaire measures are presented in Table 2.

**Table 2:** Mean and SD scores of generalized self‐efficacy, Pittsburgh sleep quality index, social support indexes, and general health questionnaire.

Variables	Mean	SD
Generalized self‐efficacy (GSE)	22.546^a^	5.403
PSQI (score)	7.67^b^	4.98
Subject sleep quality (score)	1.94	1.23
Sleep latency (min)	24.00	12.11
Sleep duration (h)	7.38	2.14
Sleep efficiency (%)	69.76	7.87
Sleep disturbance (score)	11.56	5.38
Use of sleep medications (score)	0.61	1.30
Daytime dysfunction (score)	2.65	1.56
SSI (score)	29.8^a^	13.94
Family support (score)	11.31	5.34
Friend support (score)	10.09	4.36
Other support (score)	8.40	4.24
GHQ (score)	51.487^b^	8.034
Somatic symptoms	15.459	4.876
Anxiety	14.981	3.894
Social dysfunction	13.901	3.543
Severe depression	12.011	3.441

^a^ Moderate range

^b^ High risk range

According to Table 3, self‐efficacy was significantly associated with higher sleep quality (OR: 4.362, CI: 1.481–12.853, *p* = 0.008), better physical health (OR: 2.103, CI: 1.041–4.248, *p* = 0.038), lower anxiety levels (OR: 2.175, CI: 2.203–3.934, *p* = 0.010), and greater social support (OR: 7.099, CI: 3.121–16.149, *p* < 0.001). Additionally, midwives demonstrated 3.558 times higher self‐efficacy compared to nurses, and self‐efficacy increased with age (OR: 1.707, CI: 1.033–3.116, *p* = 0.043).

**Table 3:** Associate factors with generalized self‐efficacy.

	*B*	SE	df	Sig.	OR adjusted	95% CI for EXP (*B*)	
						Lower	Upper
Age ≥ 30	0.533	0.308	1	0.043	1.705	1.033	3.116
Profession (midwives)	1.269	0.509	1	0.013	3.558	1.312	9.646
Shift duration (one shift)	−0.091	0.335	1	0.785	0.913	0.474	1.759
Sleep quality ≤ 5 (good sleep quality)	1.473	0.551	1	0.008	4.362	1.481	12.853
General health quality ≤ 23	0.498	0.856	1	0.561	1.645	0.307	8.804
Somatic symptoms	0.743	0.359	1	0.038	2.103	1.041	4.248
Anxiety	0.777	0.302	1	0.010	2.175	1.203	3.934
Social dysfunction	0.483	0.312	1	0.122	1.621	0.879	2.988
Severe depression	0.475	0.315	1	0.132	1.608	0.867	2.980
Social Support	1.960	0.419	1	0.000	7.099	3.121	16.149
Other support	0.972	0.213	1	0.000	2.645	1.741	4.018
Family support	1.265	0.240	1	0.000	3.544	2.216	5.667
Friend support	0.431	0.295	1	0.144	1.539	0.863	2.745
Constant	−8.742	1.558	1	0.000	0.000		

## 6 Discussion

The purpose of this study was to identify the factors associated with self‐efficacy among medical staff. The findings indicated that the mean general self‐efficacy score was 22.546 ± 5.403, reflecting a moderate level of self‐efficacy [19], in line with previous studies [26]. However, contrasting results exist; for example, Tsai et al. found higher levels of self‐efficacy among nurses [27], possibly due to differences in study populations and work conditions. Tsai's study included only nurses with more than 1 year of work experience and did not distinguish between day and night shifts [28], while our study encompassed a broader group, including nurses with rotating shifts and varying experience levels, factors that may have influenced self‐efficacy outcomes.

In addition to participant characteristics, differences in hospital settings, measurement tools, and study timing further contribute to variability in findings. While some studies have reported low self‐efficacy among hospital staff [29], others—particularly those conducted during the COVID‐19 pandemic—have observed moderate to high scores [26]. Different studies use various self‐efficacy measurement tools, some of which provide more comprehensive assessments of confidence in professional tasks, while others focus on generalized self‐efficacy. Moreover, variations in hospital environments, including staffing ratios, patient load, and institutional policies, may contribute to the observed discrepancies in self‐efficacy scores.

Age was another factor found to be significantly associated with self‐efficacy in our study (mean age = 33.31 years, *p* = 0.043). Although some studies have not identified a strong link between age and self‐efficacy [30], it is a well‐established fact that maturity and productivity influence how individuals think and perceive their ability to perform tasks, which, in turn affects their self‐efficacy [11]. Generally, as people grow older, they tend to develop more refined problem‐solving and decision‐making skills, which likely contribute to higher self‐efficacy levels [31]. Additionally, our findings indicated that self‐efficacy levels were 1.269 times higher among midwives compared to nurses. This difference may be attributed to the nature of the midwifery profession, where practitioners often work in settings that afford them greater autonomy in patient care decisions [32], Such autonomy may foster a stronger sense of professional confidence. Furthermore, midwifery education typically places a stronger emphasis on independent decision‐making, which may contribute to further enhancing confidence levels [33].

Among the psychological and health‐related factors assessed, social support showed the strongest positive association with self‐efficacy. This finding aligns with earlier research highlighting social support as a critical element in managing stress, enhancing resilience, and boosting professional confidence [34]. Our results further emphasize that individuals who received consistent support from both institutional systems and personal networks exhibited significantly higher self‐efficacy levels. Structured supports—such as mentoring programs, leadership encouragement, and well‐being initiatives—can thus play an important role in fostering a high‐functioning and confident healthcare workforce [35].

Similarly, self‐efficacy was found to be significantly associated with sleep quality (OR: 4.362; *p* = 0.008), an increasingly recognized issue among medical staff. Sleep disturbances are common in this population; for example, one study reported that 38.7% of 4245 healthcare workers experienced sleep disturbances [36]. Inadequate sleep impairs cognitive functions like focus and decision‐making, which are crucial for maintaining self‐efficacy in high‐pressure and demanding medical environments [37]. Previous studies support these findings, showing that poor sleep negatively impacts job performance and quality of life among nurses [38]. Our results reinforce the value of interventions aimed at improving sleep hygiene, which could in turn enhance professional efficacy and well‐being. Healthcare institutions should consider policies and interventions that promote better sleep hygiene among medical staff to ensure optimal job performance and patient care.

In addition to sleep, general health—including both physical and mental dimensions—was positively associated with self‐efficacy, particularly regarding anxiety symptoms. This is consistent with previous research suggesting that better general health contributes to higher self‐efficacy [39, 40, 41]. For example, Simonetti's study found that improvements in sleep and anxiety were accompanied by increases in self‐efficacy [41]. Our results align with this, showing a notable link between overall health status and self‐efficacy, particularly in relation to anxiety and physical complaints. Nonetheless, reports on the general health of medical staff are mixed. While Hu et al. [42] noted that general health is at risk (mean score: 51.49), Najafi et al. reported that nearly half of their participants had poor health [43], whereas Niazi et al. found relatively good health among staff [44]. These discrepancies may reflect contextual differences such as workplace culture, managerial support, and staffing levels. These results highlight the importance of promoting self‐efficacy as a potential strategy to enhance both the physical and mental well‐being of healthcare professionals.

Stress was another key variable in our study, with participants reporting high levels of stress and anxiety—findings consistent with numerous other studies that have consistently reported elevated stress levels among healthcare professionals, particularly nurses [45, 46, 47]. Chronic stress can lead to negative behaviors and attitudes toward oneself, work, family, and patients, ultimately resulting in decreased productivity, absenteeism, ethical concerns, and job dissatisfaction. Previous research has also highlighted a strong link between self‐efficacy and overall well‐being. Dadipour et al. demonstrated that an adequate level of self‐efficacy positively influences all aspects of nurses' general health [15]. Additionally, Fatehi et al. identified stress as a critical factor affecting nurses' self‐efficacy [48], while Kim's study established a relationship between stress‐coping mechanisms and self‐efficacy [26, 49].

These negative outcomes often stem from sustained psychological strain. Interventions targeting workplace stress, alongside efforts to enhance self‐efficacy, could be effective in improving mental health and overall job performance [47]. Given these findings, reducing workplace stress and strengthening self‐efficacy through targeted interventions could significantly enhance the overall well‐being and job performance of medical staff.

This study further contributes to existing literature by comprehensively examining the correlation between self‐efficacy and various factors, including demographic characteristics, sleep quality, general health, and social support. While previous studies have primarily focused on isolated factors such as spiritual intelligence, organizational support, or resilience [50, 51, 52], our research provides a more holistic perspective by integrating multiple determinants of self‐efficacy. This broader approach offers a deeper understanding of how various personal and professional factors collectively influence self‐efficacy among medical staff, reinforcing the importance of multifaceted interventions to improve confidence, well‐being, and performance in healthcare settings [53].

However, this study has a limitation in that it did not examine other potential factors that may influence self‐efficacy, such as personality traits, job satisfaction, or workplace stress management strategies. Additionally, as a descriptive and cross‐sectional study, it does not establish causality between the variables examined. For example, while a relationship was observed between self‐efficacy and sleep quality, it remains unclear whether higher self‐efficacy leads to better sleep or whether better sleep enhances self‐efficacy—an issue that cannot be resolved within the scope of this study design.

Future research should address these limitations by incorporating a broader range of influencing factors and employing longitudinal or experimental designs to determine causal relationships. Moreover, interventions aimed at enhancing self‐efficacy, particularly among nurses and midwives, should be designed and implemented to improve both individual well‐being and overall job performance in medical settings.

## 7 Conclusion

This study revealed that Iranian medical staff exhibited poor sleep quality, moderate self‐efficacy levels, low social support, and general health risks. Self‐efficacy was positively associated with better sleep quality, improved physical health, lower anxiety levels, and stronger social support. These findings underscore the urgent need for targeted interventions to enhance the psychological well‐being and coping strategies of healthcare professionals, especially those caring for patients and pregnant women. A comprehensive approach—combining systemic support, early risk identification, and resource mobilization—is essential for strengthening self‐efficacy. Practical strategies may include mindfulness training, sleep hygiene programs, social support initiatives, and stress management interventions. Integrating self‐efficacy development into healthcare policies and professional training can foster a more resilient and effective workforce, ultimately leading to improved patient care and outcomes.

## Author Contributions

**Dara Rasoal:** conceptualization, methodology, investigation, writing – original draft, writing – review and editing, and validation. **Zahra Karimian:** conceptualization, data curation, investigation, supervision, writing – original draft, writing – review and editing, validation, visualization, and methodology. **Zahra Javanbakht:** writing – review and editing, writing – original draft, methodology, investigation, and conceptualization. **Kamran Vafaee:** conceptualization, methodology, data curation, investigation, writing – original draft, writing – review and editing. **Mina Abbasi:** conceptualization, investigation, methodology, writing – original draft, writing – review and editing. **Arezoo Haseli:** conceptualization, investigation, methodology, software, formal analysis, funding acquisition, visualization, project administration, resources, supervision, writing – original draft, writing – review and editing.

## Ethics Statement

This study was a research project approved by the Ethics Committee of the Research Deputy at Kermanshah University of Medical Sciences in Kermanshah (approval code: IR. KUMS. REC.1403.183).

## Consent

All participants were fully informed about the study and provided their informed consent to participate.

## Conflicts of Interest

The authors declare no conflicts of interest.

## Transparency Statement

The authors affirm that this manuscript is an honest, accurate, and transparent account of the study being reported; that no important aspects of the study have been omitted; and that any discrepancies from the study as planned (and, if relevant, registered) have been explained.

## Data Availability

The data supporting the findings of this study are available within the article or from the corresponding author upon reasonable request.
